# A Comparative Study of Problem-Based Learning and Traditional Approaches in College English Classrooms: Analyzing Pedagogical Behaviors Via Classroom Observation

**DOI:** 10.3390/bs10060105

**Published:** 2020-06-26

**Authors:** Shifang Tang, Manli Long, Fuhui Tong, Zhuoying Wang, Henan Zhang, Kara L. Sutton-Jones

**Affiliations:** 1 Center for Research & Development in Dual Language & Literacy Acquisition, Department of Educational Psychology, College of Education and Human Development, Texas A&M University, College Station, TX 77843, USA; fuhuitong@tamu.edu (F.T.); ustop2013wzy@tamu.edu (Z.W.); henan@tamu.edu (H.Z.); kara.sutton@tamu.edu (K.L.S.-J.); 2 Department of Educational Psychology, College of Education and Human Development, Texas A&M University, College Station, TX 77843, USA; 3 College of International Education, Hubei University of Technology, Wuhan 430068, China; 20020020@hbut.edu.cn

**Keywords:** pedagogical behaviors, classroom observation, EFL

## Abstract

In this study, we described and compared an English as a foreign language (EFL) teacher’s pedagogical behaviors in traditional and problem-based learning (PBL) classroom settings in a Chinese university. In spring 2019, we collected six 45-min videos, three in each condition, covering three modules: (a) warm-up and vocabulary, (b) essay structure, and (c) writing. The analyses of the teacher’s pedagogical behaviors and her interaction with students indicated that the instructor spent most of the instructional time delivering higher-order thinking content in both traditional and PBL classes. The teacher’s activity structure influenced students’ communication mode. Although the instructor provided students with more group discussion activities in the PBL classroom, lecturing was observed to be the primary delivery method in both classes. These results suggest that the application of PBL strategies in the EFL classroom did not significantly restructure the teacher’s pedagogical behaviors, and thus, failed to achieve the goal of providing students with more opportunities for improving their expressive English language proficiency. These findings underscore the need to develop an effective PBL-related curriculum and professional development opportunities for EFL teachers to effectively implement the PBL approach in the classroom.

## 1. Introduction

Over the last few decades of intensified globalization, English has been adopted as an international language [1] and attracted increased attention from educators and learners [2]. In China, English proficiency is considered an essential skill for students from elementary school to university. At the university level, all non-English majors are required to take at least one year of College English (CE) [3,4]. The newly formulated *Guidelines for College English Teaching* [5] emphasized that College English should cultivate students’ language usability, enhance their intercultural awareness and communicative capability, to develop students’ autonomous learning capability to meet their needs in their future study, life, job, and communication. The *Guidelines* recommended the use of collaborative, inquiry-based instructional methods, and approaches to enlighten and inspire students, which emphasize a transition from teacher-centered to student-centered instruction and from passive to active learning [3]. To facilitate CE learning in English as a foreign language (EFL) contexts, where students usually lack exposure to the target language, researchers suggested that problem-based learning (PBL) is a practical approach to providing students with opportunities to demonstrate their knowledge and skills in meaningful contexts [6] and to foster students’ autonomous learning capability [7,8].

PBL is a pedagogical approach that has been widely applied in multiple educational contexts to support students’ problem-solving skills [9]. In China, PBL has been studied extensively across various disciplines, including medicine [10,11], engineering [12,13], and Chinese–English bilingual education [14,15,16]. However, very little empirical evidence of the effectiveness of PBL in CE has been reported [17]. Moreover, based on our review of the literature below, the findings are also contradictory regarding the effectiveness of PBL in CE education in China. These findings lead to a critical question: What are the current instructional practices in CE classrooms? However, documented observations of teachers’ pedagogical behaviors and evaluation of their implementation of PBL are limited. This gap in the Chinese CE education field should be addressed with much-needed empirical studies that evaluate the instructional practice and effectiveness of PBL application in CE classrooms. Therefore, in this study, we introduced a multi-dimensional and multi-categorical classroom observation protocol. Using the protocol, we then described and compared how a CE teacher allocated her instructional time and the instructional behaviors she utilized in two different approaches: Traditional and PBL. We examined the use of languages, strategies, communication mode, language content, and type of instruction.

## 2. Literature Review

In this section, we present the theoretical background of PBL and review empirical studies of PBL implementation and its effectiveness in EFL contexts. We then focus on the effectiveness of PBL on college students’ English language learning in the context of Mainland China.

### 2.1. PBL Theoretical Background

Developed from Vygotsky’s [18] theory of social constructivism, PBL is a student-oriented instructional approach [19,20,21]. PBL is learner-centered, providing a stimulus for learning by confronting students with problems from practice [22], and encouraging learners to apply knowledge and skills to develop a viable solution to a defined problem [23]. PBL was first applied in the field of medical education in the early 1960s [24,25], and later expanded and applied in other subject areas [20,26,27,28].

PBL emphasizes the integration of real-world problem-solving, critical thinking, and self-regulated active learning [29] via group discussion and peer collaboration [21]. In a PBL classroom, students are encouraged to decide on the problem and collectively generate objectives for their self-directed learning, while the teacher serves as a facilitator, rather than a content provider, to engage students to explore the content material [30,31,32,33]. Ali [19] suggested that in settings where PBL approach is used, students must take responsibility for their own learning, and teachers should act as facilitators or guides to encourage students to construct and seek meaning and solutions.

Over the last century, there has been a paradigm shift in instructional methodology from a teacher-centered approach toward a student-centered approach such as PBL that emphasizes the learner’s critical role in the learning process [34]. Researchers have found that students benefit more from student-centered instruction, compared with traditional lecture-based teaching. For example, PBL increases active participation and motivation in learning [31,35], encourages the development of problem-solving and inquiry skills [23,36], makes learning more relevant to the real world [29,37], and engages students in self-regulated learning [38].

### 2.2. PBL in China’s College EFL Classrooms: A Research Gap

The PBL approach was introduced into China in the late 20th century [39] and soon attracted the interest of educators and researchers in the field of EFL education [40]. We conducted a comprehensive review of the literature on the application of PBL in EFL classrooms in Chinese higher education in the recent 10 years. The review covered both English and Chinese databases, including Education Source, Education Resources Information Center (ERIC), and China National Knowledge Infrastructure (CNKI). The search revealed that a majority of the studies focused on a conceptual basis, theoretical application, practical significance, challenges, and recommended improvement strategies for PBL [41,42,43,44,45]. However, there was limited empirical research that investigated the application of PBL in EFL classrooms in Chinese higher education. We were able to identify eight empirical studies using the PBL approach [39,46,47,48,49,50,51,52], and our syntheses and critiques of these studies are presented from three perspectives: English language proficiency, critical thinking skills, and the quality of implementing PBL.

First, seven of the empirical studies investigated the influence of the PBL teaching model on the development of students’ English language acquisition [39,46,48,49,50,51,52]. For instance, Zhang and Lin [50] and Chen [46] confirmed the positive impact of PBL on college students’ English language proficiency, as measured by the *National College English Test Band 4 (CET-4)*. Zheng [51] and Li [48] found similar results using a researcher-designed instrument. Moreover, Wang [39] and Zhang [49] specifically examined college students’ English oral language development as the result of participating in PBL activities. They showed that compared with traditional instruction, PBL resulted in better English oral language skills development. However, two other studies comparing PBL and traditional approaches indicated no difference between the two conditions in supporting college students’ English language development [52] and vocabulary and syntax acquisition [49].

One of the main benefits of PBL for students is the development of critical thinking skills, something that has been studied by researchers. For example, four studies incorporated the *California Critical Thinking Disposition Inventory (CCTDI)* [53] and its revised version [46,54] to measure how PBL impacts college students’ critical thinking dispositions and skills in learning English [46,47,50,52]. Ding [47] found that PBL significantly improved college students’ critical thinking dispositions, including truth-seeking, open-mindedness, analyticity, systematicity, critical thinking, self-confidence, inquisitiveness, and cognitive maturity. The three other studies [46,50,52] provided evidence that PBL supported students’ development of critical thinking dispositions, including openness, analysis, and fairness, and critical thinking skills, such as analyzing and explaining. Zhang and Lin [50] and Chen [46] found that PBL also benefited students’ critical thinking dispositions of truth-seeking, self-confidence, and maturity, and critical thinking skills of interpreting and evaluating.

We were also interested in knowing whether and how the researchers of the included studies monitored the quality of PBL implementation in the classroom. Eight studies reported the quality of the PBL implementation [39,46,47,48,49,50,51,52]. Although various methodological approaches were used in these studies, such as interviews [39,46,50,52], student evaluation questionnaires [48,49,51], and classroom observation field notes [47], detailed information, such as how interviews or observations were conducted and how codes were established to analyze observation or interview data, were not provided. These methodological details are critical to the validity of research results [55].

Taken together, the recent body of research indicated mixed findings regarding the impact of PBL on college students’ English language proficiency and their critical thinking skills in English classrooms. These findings raise concerns regarding the quality of the application of PBL in EFL classrooms. Moreover, none of them provided details of how the researchers evaluated the quality or how they analyzed the evaluation data. This lack of detail suggests the use of subjective judgment on the evaluation of PBL quality, and therefore significantly undermines the validity of their findings.

Without evidence from direct classroom observation that reveals what happened in the PBL classrooms, one cannot objectively determine whether and how much PBL was implemented, which would be the first step of evaluating the effectiveness of PBL. Thus, there is a dire need to conduct observation studies with a comprehensive and reliable instrument that describes and quantifies the quality of the PBL implementation. Therefore, the purpose of this study was two-fold. First, we introduced a multi-dimensional and multi-categorical classroom observation instrument that was developed from sound pedagogical theories and was validated and applied in numerous studies in English as a second language (ESL), EFL, and bilingual education contexts. Second, we used this instrument to describe and compare the teacher’s time allocation of pedagogical behaviors between PBL and traditional classrooms of first-year college students were learning English. Specifically, we examined the time allocation in the following dimensions of instruction: (a) Language content, (b) activity structure, (c) communication mode, (d) language of instruction (teacher instructional language and student response language), (e) ESL strategies, and (f) physical group. Three research questions guided our study:

1. Does teacher’s time allocation of pedagogical practices differ between PBL and traditional classes in the warm-up and vocabulary module?
2. Does teacher’s time allocation of pedagogical practices differ between PBL and traditional classes in the essay structure module?
3. Does teacher’s time allocation of pedagogical practices differ between PBL and traditional classes in the writing module?

## 3. Method

### 3.1. Research Context and Participants

This research was conducted in a public university located in the central part of China. It is a tier-one, comprehensive university that was established in 1952. The university is now serving 18,801 full-time undergraduate students, 6334 graduate students, and 950 international students with 2110 faculty and staff members. In this study, 57 freshmen were randomly assigned to two College English II classrooms by the university administration. These two classes were randomly assigned to the participating instructor (one of the co-authors), who randomly selected one class to receive PBL instruction and one to receive traditional instruction. The randomization process resulted in 29 students in the PBL class and 28 in the traditional class. The instructor had been using the PBL approach for six years in her classrooms and sought an evaluation from our research team on her application of the PBL approach.

### 3.2. Description of the College English II Course

The College English II course was a two-credit-hour core course designed to develop students’ general English language proficiency. Students met with the instructor two times a week, with each class lasting 90 min for 16 weeks in spring, 2019. The course was delivered in two phases. The first phase was a five-week, textbook-based section, which included three book units: Unit 2 of Book 2, and Units 2 and 3 of Book 3, all from the *New Horizon College English* (third edition, MOE-approved textbook for College English). Each unit was composed of three modules: (a) Warm-up and vocabulary, (b) essay structure, and (c) writing, with each module lasting 90 min. The PBL approach was only applied in the first phase. The second phase was an 11-week, exam-oriented section, in which the instructor’s main goal was to prepare students for the *College English Test Band 4 (CET-4).* Four modules, i.e., listening, reading, translation, and writing, were included to reflect the sections of the test. The current study focused on the comparison between PBL and traditional classes that were taught by the same teacher during Unit 2 of Book 2 of the first phase.

PBL instruction in the College English II classrooms consisted of:

1. Module 1: Warm-up and vocabulary. The instructor taught vocabulary and new content knowledge by asking questions. She provided students with opportunities to discuss within a small group. Students responded to the questions and received evaluation and cognitive feedback from the teacher.
2. Module 2: Essay structure. The instructor divided students into three large groups. The students were requested to discuss the main idea and structure of the text. After the discussion, a representative of each group presented their findings.
3. Module 3: Writing. The instructor provided students with opportunities to discuss the structure and writing techniques of corresponding reading materials. A representative of each group presented their ideas to the whole class and received feedback from the instructor. The instructor introduced one or two writing samples and evaluated their strengths and weaknesses. In the end, the teacher summarized writing techniques and assigned students a writing template for practicing writing after class.

### 3.3. Traditional Classroom

Instruction in the traditional College English II classrooms included:

1. Module 1: Warm-up and vocabulary. The instructor gave direct lectures to introduce the content knowledge of the unit and new vocabulary.
2. Module 2: Essay structure. The instructor spent most of the instructional time delivering lectures. Students were provided with a couple of opportunities to read the text.
3. Module 3: Writing. Lecturing was the main instructional approach. The instructor analyzed writing materials, gave lectures on writing techniques, and evaluated some writing samples. Students were required to practice writing in the class.

### 3.4. Observation Instrument

The four-dimensional Transitional Bilingual Observation Protocol (TBOP) [56] was used for classroom observation in this study. TBOP is a multi-dimensional and multi-categorical classroom observation instrument developed from the four-dimensional pedagogical theory by Lara-Alecio and Parker [56]. It was designed to capture a comprehensive picture of teachers’ instructional delivery and students’ engagement. The original four dimensions are: (a) Language content, (b) activity structure, (c) communication mode, and (d) language of instruction (teacher instructional language and student response language). Two more domains, ESL strategies and physical group, were added in the protocol to further understand instructional patterns in the ESL classrooms [57]. To be specific, in the domain of language content, there are four categories: (a) Social routines (e.g., greetings), (b) academic classroom routines (e.g., preparation of academic task), (c) light cognitive (e.g., reviewing previous knowledge), and (d) dense cognitive (e.g., critical thinking, new vocabulary learning). The domain of communication mode includes two receptive language modes (listening and reading), two expressive ones (verbal and writing), and the combination of these modes (e.g., listening and speaking, reading and writing). The activity structure domain is the interaction between teacher behavior (e.g., directing, lecturing, evaluating, observing) and students’ expected response (e.g., listening, performing, discussing, answering, cooperative learning). In the domain of language of instruction, students’ first (L1) and second language (L2) can be used in the following ways: (a) Content introduced in L1, (b) use L1 to introduce L2, (c) use L1 to further clarify and support L2, and (d) content presented in L2. The domain of ESL strategies, which is teachers’ use of instructional approach to scaffold students’ learning of English language, includes nine specific strategies (e.g., questioning strategies [QS], academic language scaffolding [ALS], advanced organizer [AO], language clarification [LC], visual scaffolding [VS], cooperative grouping [CG], and manipulative realia [MR]). In the domain of physical group, there are five specific categories: (a) Total class, (b) large group, (c) small group, (d) pairs, and (e) single (see [55] for a more detailed description of TBOP). TBOP has been applied in the fields of bilingual education [57,58], ESL education [55,59], and EFL education [60,61]

### 3.5. Data Collection

The lectures were recorded during the spring semester of 2019 by the instructor for students to review when they were absent or needed extra support to understand the lectures. The videos were blurred when sent for analysis in this study. Three units of the College English course were observed in both PBL and traditional instruction classes; each unit consisted of three modules: (a) Warm-up and vocabulary, (b) essay structure, and (c) writing. Two classrooms were observed on the same unit and module. Each module lasted 45 min. Therefore, the total length of observation for six videos was 270 min. The videos were coded later by two graduate research assistants who received intensive TBOP coding training and achieved interrater reliability above a substantial level (Gwet AC_1_ > 0.61) [62]. During the coding process, coders coded every 20-s video clip during the first, third, fifth, and seventh 5-min intervals. In each 20-s video clip, the coders used the TBOP coding sheet to document the occurrence of the categories under each domain. For example, in a 20-s video clip, the coder documented QS (in the domain of ESL strategies), SG (in the domain of physical group), ask/ans (in the domain of activity structure), au-ver (in the domain of mode), dns cog (in the domain of language content), L2 (in the domain of language of instruction (teacher)), and L2 (in the domain of language of instruction (student)). This coding set can be interpreted that during this 20-s video clip, the teacher was observed to use a questioning strategy to ask a higher-order thinking question in English, and a student responded in English. Each video contained 60 coding sets. Therefore, in this study, there were 360 coding sets included in the analysis. The rationale and details of coding protocol and instrument can be found in [55,59].

### 3.6. Data Analysis

The data collected via TBOP coding were frequency data in nature. Therefore, a chi-square test of homogeneity was conducted to determine if there were any significant differences in terms of teacher’s instructional time allocation between PBL and traditional classes. The analyses were conducted by module and by domain. For example, in Module 1 warm-up and vocabulary, for the domain of language content, we performed the chi-square test to identify if the frequency of each category (i.e., social, academic routine, light cognitive, and dense cognitive content) was homogeneous between PBL and traditional classes. Moreover, given the multiple categories of each domain of TBOP, a post hoc test using the standardized residual method suggested by Beasley and Schumacker [63] was conducted where a significant difference was identified. Cramer’s V was reported as effect size in this study, and the guidelines from Rea and Parker [64] were adopted for reporting the magnitude of effect size: <0.10, negligible; 0.10–0.20, weak; 0.20–0.40, moderate; 0.40–0.60, relatively strong; 0.60–0.80, strong; 0.80–1.00, very strong.

## 4. Results

### Quantitative Analysis with the TBOP Instrument

After the initial transcribing and analysis of the classroom observation videos, to further evaluate the pedagogical behaviors of the instructor, we analyzed classroom discourses from a multi-dimensional perspective. In this section, we present the results of the analyses conducted to compare the instructor’s pedagogy practice between PBL and traditional classes to address each research question. Chi-square analysis was repeated in all six domains in all three instructional modules: (a) Warm-up and vocabulary, (b) essay structure, and (c) writing.

RQ #1: Does teacher’s time allocation of pedagogical practices differ between PBL and traditional classes in the warm-up and vocabulary module?

In the first module, no significant difference was identified regarding the instructor’s time allocation in all six domains (i.e., language content, ESL strategies, language of instruction (teacher and student), activity structure, mode, and physical group) between the PBL and traditional classes (see Table 1). Since there was no significant main effect, no post hoc analysis is needed. In the domain of language content, we observed that the instructor allocated more than 86% of the instructional time in dense cognitive content to deliver new or higher-order thinking content to the students in both the PBL and traditional classes. In the domain of ESL strategies, we observed that the teacher most frequently used the visual scaffolding strategy (43.3% in PBL, 55% in the traditional class). The instructor employed other ESL strategies, such as academic language scaffolding (in both classes) and cooperative/collaborative strategies (PBL only), but the application of these strategies was minimal, less than 10%. It was worth noting that for more than one-third of the instructional time, no strategy was utilized.

In the domain of language of instruction (teacher), the instructor spent the majority of her time delivering instruction in English (85% in PBL, 76.7% in the traditional class). In the domain of language of instruction (student), students in both classes were given very limited time to respond in English (16.7% in PBL, 8.3% in the traditional class). Furthermore, students in both classes were silent during most of the instructional time (81.7% in PBL, 90% in the traditional class).

In the domain of activity structure, the instructor allocated almost half of the instructional time to lecture/listing (45% in PBL, 56.7% in the traditional class). Evaluate/performance was the second most frequently used instructional activity (26.7% in PBL, 11.7% in the traditional class). Other interactive instructional activities, such as ask/answer and ask/performance, were seldom employed, with less than 5%. In the domain of mode, the instructor most frequently engaged students in a receptive language mode, such as listening (65% in PBL, 68.3% in the traditional class), while minimal opportunities were provided to involve students in both receptive and expressive language mode, such as speaking/listening (5% in both classes) and listening/speaking (less than 9% in both classes). In the domain of physical group, the instructor spent most of her time in whole-class instruction (91.7% in PBL, 93.3% in the traditional class); she rarely used one-on-one student interaction (8.3% in PBL, 6.7% in the traditional class).

RQ #2: Does teacher’s time allocation of pedagogical practices differ between PBL and traditional classes in the essay structure module?

In the module of essay structure, statistical significant differences were identified regarding the instructor’s time allocation in four domains: ESL strategies (χ^2^(3) = 28.644, *p* < 0.001, Cramer’s V = 0.489), physical group (χ^2^(1) = 4.138, *p* = 0.042, Cramer’s V = 0.186), activities structure (χ^2^(6) = 30.768, *p* < 0.001, Cramer’s V = 0.506), mode (χ^2^(6) = 20.815, *p* < 0.001, Cramer’s V = 0.416). Post hoc analysis was conducted for those domains, with standardized residual reported. No significant difference was identified in the domains of language content and language of instruction (teacher and student) between the PBL and traditional classes (see Table 2).

In the domain of ESL strategies, visual scaffolding strategy was observed to be the most frequently used (40% in PBL, 33.3% in the traditional class). The instructor employed cooperative/collaborative strategies in the PBL class only (25% in PBL, 0% in the traditional class, *p* = 0.01). The instructor used significantly fewer strategies in the traditional class (28.3% in PBL, 66.7% in the traditional class, *p* = 0.03).

In the domain of activity structure, the instructor allocated almost half of the instructional time to lecture/listening (51.7% in PBL, 45% in the traditional class). The results of post hoc analysis indicated that the instructor allocated significantly more time to evaluate/cooperation activities in the PBL class (21.7% in PBL, 0% in the traditional class, *p* = 0.01) and more to evaluate/performance in the traditional class (8.3% in PBL, 33.3% in the traditional class, *p* = 0.04). In the domain of mode, the instructor allocated the majority of instructional time to involving students in a receptive language mode, such as listening (65% in PBL, 68.3% in the traditional class). The results of post hoc analysis indicated that students in both the PBL and traditional classes were provided opportunities of practicing both receptive and expressive language skills, such as speaking/listening (33.3% in PBL, 11.7% in the traditional class, *p* = 0.07) and listening/speaking (3.3% in PBL, 16.7% in the traditional class, *p* = 0.11). In the domain of physical group, the instructor spent most of her time in whole-class instruction (96.7% in PBL, 93.3% in the traditional class). No one-on-one interaction was observed in the PBL class (0% in PBL, 6.7% in the traditional class).

In the domain of language content, the instructor spent more than 90% of the instructional time in dense cognitive content to deliver new or higher-order thinking content to the students in the both PBL and traditional classes. In the domain of language of instruction (teacher), the instructor spent the majority time delivering instruction in English (85% in PBL, 66.7% in the traditional class). In the domain of language of instruction (student), students in both classes were silent during the majority of the instructional time (63.3% in PBL, 71.7% in the traditional class). However, PBL students received more opportunities to practice their oral language in English (36.7% in PBL, 27.7% in the traditional class).

RQ #3: Does teacher’s time allocation of pedagogical practices differ between PBL and traditional classes in the writing module?

In the writing module, we identified statistically significant differences regarding the instructor’s time allocation in three domains: ESL strategies (χ^2^(3) = 17.971, *p* < 0.001, Cramer’s V = 0.387), physical group (χ^2^(1) = 4.138, *p* = 0.042, Cramer’s V = 0.186), and language of instruction (teacher) (χ^2^(3) = 20.745, *p* < 0.001, Cramer’s V = 0.416). A marginal significant difference was found in the domain of mode (χ^2^(7) = 13.219, *p* = 0.067, Cramer’s V = 0.332). There were no significant differences the domains of activity structure, language content, and language of instruction (student) between the PBL and traditional classes (see Table 3).

In the domain of ESL strategies, the instructor employed a visual scaffolding strategy more frequently in the traditional class than the PBL class (13.3% in PBL, 41.7% in the traditional class, *p* = 0.04). Cooperative/collaborative strategies were used in both the PBL and traditional classes (20% in PBL, 28.3% in the traditional class). Moreover, in 63.3% of the instructional time in the PBL class, the instructor did not employ any ESL strategies (28.3% in the traditional classes, *p* = 0.05). In the domain of physical group, the instructor spent all of her time in whole-class instruction in the traditional class and 93.3% in the PBL class. Very limited one-on-one interaction was observed in the PBL class (6.7% in PBL, 0% in the traditional class).

In the domain of activity structure, lecture/listing was observed to be the most frequently used (36.7% in PBL, 46.7% in the traditional class). Evaluate/performance (25% in PBL, 10% in the traditional class) and evaluate/cooperation (11.7% in PBL, 21.7% in the traditional class) were also frequently observed activities in both classrooms. In the domain of mode, the instructor allocated half of the instructional time in involving students in a receptive language mode, such as listening (46.7% in PBL, 56.7% in the traditional class). The instructor spent one-third of the instructional time in both receptive and expressive communication modes, such as speaking/listening (30% in PBL, 33.3% in the traditional class). Although it is the writing module, no writing activity was observed in the traditional class, while in the PBL class, the instructor spent 15% of instructional time on writing activities (*p* = 0.04).

In the domain of language content, the instructor allocated more than 90% of the instructional time in dense cognitive content in both the PBL and traditional classes. In the domain of language of instruction (teacher), the instructor spent more time delivering instruction in English in the traditional class (53.3% in PBL, 90% in the traditional class, *p* = 0.09). Moreover, the instructor was silent 28.3% of the instructional time in the PBL class (8.3% in the traditional class, *p* = 0.07). In the domain of language of instruction (student), students in both classes were silent during the majority of the instructional time (65% in PBL, 61.7% in the traditional class), and students in both classrooms had similar opportunities to practice their oral language in English (35% in PBL, 38.3% in the traditional class).

## 5. Discussion

The purpose of the present study was to describe and compare time allocation of an EFL instructor’s pedagogical behavior and interaction with students between her PBL and traditional classes in three lecture sessions: (a) Warm-up activities, (b) essay structure, and (c) writing. Via a multi-dimensional and multi-categorical classroom instrument, we examined the instructor’s pedagogical behavior across six dimensions: Language content, activity structure, communication mode, language of instruction (teacher instructional language and student response language), ESL strategies, and physical group.

### 5.1. Language Content and Language of Instruction

Descriptive and inferential analysis of language content and language of instruction (teacher and student) revealed similarities and differences in the PBL and traditional classrooms. First, except for the teacher language of instruction in the module of writing, the PBL classroom did not differ from the traditional classroom in the domains of language content and language of instruction (teacher and student). The results indicated that the instructor allocated more than 85% of her instructional time to delivering dense cognitive content in all three lecture sessions in both the PBL and traditional classes. The results illustrated that the instructor managed to facilitate students’ mastery of new vocabulary, sentence structure, grammatical points, as well as develop students’ ability in higher-order thinking, logical reasoning, and problem-solving skills in both classrooms.

Second, during more than 50% of the instructional time, the instructor taught in English and exposed students to an English-speaking environment in both classrooms. However, she provided few opportunities for students to practice oral English, which can be observed in the domain of language of instruction (student). The students in both conditions were silent during more than 60% of the instructional time in all three lecture sessions. The pattern was even more evident in the module of warm-up activities, which was supposed to foster a positive learning environment for students to practice and experiment with language [65] and lower student anxieties about using the language [66]. The finding was consistent with previous research that it is hard to gauge the quality of students’ performance in the PBL classroom when the students were not provided opportunities to talk [67]. Although the instructor raised questions from time to time, it seemed that she did not expect all students to respond or involve the students in interacting with their peers or herself to produce complex language content. The highly teacher-centered instruction used in the warm-up and other sessions in both conditions did not differentiate her PBL instruction from the traditional instruction. This observation was similar to previous research indicating that students cannot benefit from PBL if PBL was poorly implemented, such as providing students with minimum participation and engagement [68,69]. Such teacher-dominated instruction with a low student response was not surprising, because previous observation studies [61,70,71] also reported that Chinese students are often quiet and passive in English-speaking classrooms. However, in order for students to develop strong English language proficiency, teachers need to provide sufficient opportunities for students to actively participate in oral discussion, peer collaboration, individual/group presentation, and writing throughout the semester.

### 5.2. Teacher Activity Structure, Student Communication Mode, ESL Strategies, and Grouping

The passive student learning echoes our finding from the analysis of the domain of Activity Structure and Communication Model, in which lecture/listen and listening were the most frequently used in both the PBL and traditional classes across the three modules, which also aligned with passive student learning observed in the domain of instructional language (student). For example, in the modules of warm-up and vocabulary and essay structure, lecture/listen (in the domain of teacher activity structure) was observed during approximately 50% of the instructional time, while listening (in the domain of student communication mode) was also observed 50% of the instructional time. The above findings were consistent with what was observed in a previous PBL study in that it was difficult for teachers to release control of their classrooms and provide students with more opportunities to explore and learn [72]. We agree that some teachers struggle with balancing their roles as an instructor and a facilitator, and at the same time provide sufficient guidance for students to develop their cognitive academic language proficiency and knowledge [30,32]. Therefore, teachers need to be well-prepared for constructing meaningful questions and effectively engaging and facilitating students’ active learning [19,73].

Significant differences were identified in the modules of essay structure and writing regarding teacher activity structure and student communication mode between PBL and traditional classrooms. In the module of essay structure, the instructor provided more opportunities to PBL students working in groups, while in the traditional class, she gave more opportunities to students to perform academic tasks. In both classrooms, she evaluated students’ learning performance and provided feedback. These findings were consistent with the observation from the domains of ESL strategies and physical group. The instructor incorporated cooperative grouping strategies in the PBL classroom, while the application of the same strategy was not observed in the traditional class. Moreover, based on the findings from the domain of physical group, the instructor evaluated students’ cooperation as a whole class and did not provide specific feedback to each group in the PBL class. Furthermore, in the module of writing, results indicated that the instructor focused more on involving students in groups to discuss writing techniques instead of having students practice writing skills. Huang, Jonassen, and Liu [74] emphasized that in PBL classrooms, students should be engaged in constructing meaningful content while working toward a solution to the problem. However, the results of the present observation showed that the desired writing opportunities were not provided by the instructor in the PBL classroom.

### 5.3. Consistencies and Discrepancies Between the Lesson Plan and Instructional Behaviors

Based on the observation, we found that there were consistencies and discrepancies between the instructor’s self-reported lesson plan and the observed instructional behaviors in the classrooms. For example, the instructor reported that she would teach new content and higher-order thinking skills in all three modules in both the PBL and traditional classrooms. Our observation indicated she spent more than 85% of her instructional time in dense cognitive content for students to learn new content and critical thinking skills. She demonstrated consistency in the language of content throughout all her teaching. Furthermore, the instructor stated that lecturing would be the major delivery method across three modules in the traditional classroom. Our observation indicated that in the traditional class, lecturing was the most frequently observed instruction activity, and listening was the most frequently observed student communication mode.

Discrepancies appeared between the PBL lesson plan and the instructor’s implementation of PBL. For example, the PBL lesson plan emphasized teacher–student and student–student interaction in the classroom across three modules. However, analysis of the classroom observational data indicated very limited interaction in the modules of warm-up activities and essay structure. The low teacher–student and student–student interactions in the PBL classroom called into question the fidelity of implementation of the lesson plan. Research has shown that PBL is an effective curricular strategy for involving students in higher-order cognitive processes and skills [75,76,77]. Obviously, the instructor’s pedagogical behaviors in the PBL classroom examined in this study failed to demonstrate these necessary characteristics of PBL for developing advanced expressive English language proficiency. Furthermore, the instructor stated that in the PBL classroom, she would provide feedback to student discussion and responses. However, our observations revealed that in the PBL classroom, the students did not receive specific feedback to their group discussion, since 90–100% of the time was spent in whole-class instruction.

### 5.4. Conclusions and Implications of PBL in College EFL Education

To conclude, using a multi-dimensional and multi-categorical classroom observation instrument, we described and compared how a university instructor allocated her instructional time in PBL and traditional classrooms. The domains explored included ESL strategies, physical group, language of instruction, language content, mode, and activity structure. Students’ passive learning that we observed in the module of warm-up in PBL classroom might relate to the teacher’s inability to adequately scaffold the students for authentic communication and to improve interpersonal and presentation skills in English, which is not surprising given that this was the first module in the first unit that she applied PBL approach in the semester. Regardless of this limitation, our findings supported that the students in the PBL classroom benefited from the PBL approach. Based on our observation, the students were approved substantial opportunities for interaction and collaboration with their peers in the module of the essay structure, which promotes creative problem-solving and higher-order thinking skills. In the same module, we observed that students were more involved in both receptive and expressive language mode (i.e., speaking and listening), which indicated in their collaborative groups, the students developed safe spaces for exchanging ideas and practicing English.

In this study, lecturing was the most frequently observed activity in the both PBL and traditional classes, which was consistent with previous studies [78,79] that lecturing is the most common form in higher education. Although researchers have suggested to combine student-centered pedagogical practices and lectures [80] to effectively implement PBL, EFL instructors need to become cognizant of patterns of teacher–student and student–student interaction in the classrooms to develop students’ higher-order thinking skills and English language proficiency. One possible way of developing such awareness is to record their own lectures and analyze the recordings via a multi-dimensional and multi-categorical instrument as we did in this study. This process can enable EFL teachers to evaluate their effectiveness and quality of PBL implementation from a critical distance. We believe that TBOP is a comprehensive and reliable classroom observation instrument for documenting teachers’ pedagogical behaviors and teacher–student interaction in college EFL settings. It helps to analyze instructional occurrences in the classroom, and therefore, can be useful for education institutions as part of their evaluation process of EFL interventions.

Furthermore, the findings in this study suggest that the instructor’s activity structure defines students’ communication mode, and students’ language mirrors their instructor’s, which is consistent with previous studies [61]. Therefore, EFL teachers need to be equipped with various interactional strategies that can encourage student discussion and exploration to improve students’ English language proficiency in solving problems. Last but not least, given the low percentage of teacher–student and student–student interactions found in PBL classes, EFL professional development programs should give special attention to fostering of teachers’ ability to design effective PBL activities and skills appropriate for implementing these particular instructional objectives by tapping into a variety of higher-order questions that can stimulate students to think and respond. There is sufficient evidence from second language acquisition studies that students’ development of advanced second language proficiency depends on rich and negotiated target language input and output [81,82]. Hu and Duan [83] suggested that well-designed professional training increased teacher use of higher-order questioning strategies in the classrooms to scaffold more productive use of English by students.

Last but not least, to further facilitate research on PBL application under EFL contexts in higher education, three methodological limitations of this study need to be recognized. First, we only observed a teacher’s application of PBL in a single university, which might not be generalizable to all EFL teachers’ application of PBL strategies at other Chinese universities where university support and intellectual profiles of instructors and students may differ from the current university. Such variation may also lead to different applications of PBL and different patterns of teachers’ pedagogical practices and interaction between students. Future research can replicate this study across different Chinese universities to verify the generalization of its findings. Some institutional factors, such as institutional policy, resources, and constraints, might be considered by the future researchers. Second, this study compared differences in pedagogical practices in PBL and traditional classes that were taught by only one teacher. Although we observed three different instruction modules in both conditions, this snapshot might not represent all teacher–student interaction in other modules. Future researchers might observe more lessons taught by one teacher or by more teachers to obtain a more comprehensive picture of how EFL teachers apply PBL in different content areas. Teacher factors, such as teachers’ motivation, personality, and professional development, can be explored as they relate to their application of the PBL approach. Finally, our feasibility of data did not allow us to explore whether and how other variables, such as teaching experience, students’ major and gender, cultural preferences, and professional development, may shape pedagogical practice and classroom discourse in the PBL and traditional classrooms. Future research is needed to understand how other teacher and student variables might impact teacher teaching and student learning in the PBL-applied EFL classrooms.

## Figures and Tables

**Table 1:** Chi-square statistics of instructor’s pedagogical behaviors in the warm-up and vocabulary module between the problem-based learning (PBL) and traditional classes.

			Condition					
			Traditional	PBL	Value	*df*	*p*	Cramer’s V
ESL Strategy	QS	N	0	2	9.031	5	0.108	0.274
		%	0.00%	3.30%				
	ALS	N	3	6				
		%	5.00%	10.00%				
	VS	N	33	26				
		%	55.00%	43.30%				
	CG	N	0	4				
		%	0.00%	6.70%				
	IT	N	0	1				
		%	0.00%	1.70%				
	NA	N	24	21				
		%	40.00%	35.00%				
Total		N	60	60				
		%	100.00%	100.00%				
Physical Group	1	N	56	55	0.12	1	0.729	0.032
		%	93.30%	91.70%				
	5	N	4	5				
		%	6.70%	8.30%				
Total		N	60	60				
		%	100.00%	100.00%				
Activity Structure	lec/lis	N	34	27	16.222	10	0.093	0.368
		%	56.70%	45.00%				
	lec/per	N	0	3				
		%	0.00%	5.00%				
	dir/per	N	1	0				
		%	1.70%	0.00%				
	led/per	N	2	0				
		%	3.30%	0.00%				
	ask/per	N	1	1				
		%	1.70%	1.70%				
	ask/ans	N	5	3				
		%	8.30%	5.00%				
	ev/per	N	7	16				
		%	11.70%	26.70%				
	obs/per	N	5	2				
		%	8.30%	3.30%				
	ev/cop	N	0	3				
		%	0.00%	5.00%				
	obs/cop	N	0	1				
		%	0.00%	1.70%				
	NA/tran	N	5	4				
		%	8.30%	6.70%				
Total		N	60	60				
		%	100.00%	100.00%				
Mode	writing	N	2	3	11.083	9	0.27	0.304
		%	3.30%	5.00%				
	aural	N	41	39				
		%	68.30%	65.00%				
	wr-au	N	3	5				
		%	5.00%	8.30%				
	re-wr	N	3	0				
		%	5.00%	0.00%				
	re-au	N	1	1				
		%	1.70%	1.70%				
	au-wr	N	2	4				
		%	3.30%	6.70%				
	au-re	N	2	0				
		%	3.30%	0.00%				
	ver-au	N	1	5				
		%	1.70%	8.30%				
	au-re-ver	N	2	0				
		%	3.30%	0.00%				
	au-ver	N	3	3				
		%	5.00%	5.00%				
Total		N	60	60				
		%	100.00%	100.00%				
Language Content	Academic	N	5	4	0.121	2	0.941	0.032
		%	8.30%	6.70%				
	Light	N	3	3				
		%	5.00%	5.00%				
	Dense	N	52	53				
		%	86.70%	88.30%				
Total		N	60	60				
		%	100.00%	100.00%				
Language of Instructional (Teacher)	L1	N	5	0	6.234	4	0.182	0.228
		%	8.30%	0.00%				
	L2	N	46	51				
		%	76.70%	85.00%				
	L1-L2	N	1	2				
		%	1.70%	3.30%				
	L2-L1	N	3	4				
		%	5.00%	6.70%				
	NA	N	5	3				
		%	8.30%	5.00%				
Total		N	60	60				
		%	100.00%	100.00%				
Language of Instructional (Student)	L1	N	1	1	1.909	2	0.385	0.126
		%	1.70%	1.70%				
	L2	N	5	10				
		%	8.30%	16.70%				
	NA	N	54	49				
		%	90.00%	81.70%				
Total		N	60	60				
		%	100.00%	100.00%				

**Table 2:** Chi-square statistics of instructor’s pedagogical behaviors in the essay structure module between the PBL and traditional classes.

			Condition					
			Traditional	PBL	Value	*df*	*p*	Cramer’s V
ESL Strategy	QS	N	0	4	26.644	3	<0.001	0.489
		%	0.00%	6.70%				
		S.R	−1.4	1.4				
	VS	N	20	24				
		%	33.30%	40.00%				
		S.R	−0.4	0.4				
	CG	N	0	15				
		%	0.00%	25.00%				
		S.R	−2.7	2.7				
	NA	N	40	17				
		%	66.70%	28.30%				
		S.R	2.2	−2.2				
Total		N	60	60				
		%	100.00%	100.00%				
Physical Group	TC	N	56	60	4.138	1	0.042	0.186
		%	93.30%	100.00%				
		S.R	−0.3	0.3				
	single	N	4	0				
		%	6.70%	0.00%				
		S.R	1.4	−1.4				
Total		N	60	60				
		%	100.00%	100.00%				
Activity Structure	lec/lis	N	27	31	30.768	6	<0.001	0.506
		%	45.00%	51.70%				
		S.R	−0.4	0.4				
	ask/ans	N	8	5				
		%	13.30%	8.30%				
		S.R	0.6	−0.6				
	ev/per	N	20	5				
		%	33.30%	8.30%				
		S.R	2.1	−2.1				
	ev/cop	N	0	13				
		%	0.00%	21.70%				
		S.R	−2.5	2.5				
	obs/dis	N	4	0				
		%	6.70%	0.00%				
		S.R	1.4	−1.4				
	obs/cop	N	0	2				
		%	0.00%	3.30%				
		S.R	−1	1				
	NA/tran	N	1	4				
		%	1.70%	6.70%				
		S.R	−0.9	0.9				
Total		N	60	60				
		%	100.00%	100.00%				
Mode	writing	N	4	0	20.815	6	0.002	0.416
		%	6.70%	0.00%				
		S.R	1.4	−1.4				
	reading	N	1	0				
		%	1.70%	0.00%				
		S.R	0.7	−0.7				
	aural	N	34	38				
		%	56.70%	63.30%				
		S.R	−0.3	0.3				
	au-wr	N	2	0				
		%	3.30%	0.00%				
		S.R	1	−1				
	au-re	N	2	0				
		%	3.30%	0.00%				
		S.R	1	−1				
	ver-au	N	7	20				
		%	11.70%	33.30%				
		S.R	−1.8	1.8				
	au-ver	N	10	2				
		%	16.70%	3.30%				
		S.R	1.6	−1.6				
Total		N	60	60				
		%	100.00%	100.00%				
Language Content	Academic	N	1	4	2.036	2	0.361	0.13
		%	1.70%	6.70%				
	Light	N	3	2				
		%	5.00%	3.30%				
	Dense	N	56	54				
		%	93.30%	90.00%				
Total		N	60	60				
		%	100.00%	100.00%				
Language of Instructional (Teacher)	L1	N	3	3	6.882	4	0.142	0.239
		%	5.00%	5.00%				
	L2	N	40	51				
		%	66.70%	85.00%				
	L1-L2	N	7	3				
		%	11.70%	5.00%				
	L2-L1	N	5	2				
		%	8.30%	3.30%				
	NA	N	5	1				
		%	8.30%	1.70%				
Total		N	60	60				
		%	100.00%	100.00%				
Language of Instructional (Student)	L1	N	1	0	2.256	2	0.324	0.137
		%	1.70%	0.00%				
	L2	N	16	22				
		%	26.70%	36.70%				
	NA	N	43	38				
		%	71.70%	63.30%				
Total		N	60	60				
		%	100.00%	100.00%				

S.R. = Standardized Residual.

**Table 3:** Chi-square statistics of the instructor’s pedagogical behaviors in the writing module between the PBL and traditional classes.

			Condition					
			Traditional	PBL	Value	*df*	*p*	Cramer’s V
ESL Strategy	QS	N	1	2	17.971	3	<0.001	0.387
		%	1.70%	3.30%				
		S.R	−0.4	0.4				
	VS	N	25	8				
		%	41.70%	13.30%				
		S.R	2.1	−2.1				
	CG	N	17	12				
		%	28.30%	20.00%				
		S.R	0.7	-0.7				
	NA	N	17	38				
		%	28.30%	63.30%				
		S.R	−2	2				
Total		N	60	60				
		%	100.00%	100.00%				
Physical Group	TC	N	60	56	4.138	1	0.042	0.186
		%	100.00%	93.30%				
		S.R	0.3	−0.3				
	single	N	0	4				
		%	0.00%	6.70%				
		S.R	−1.4	1.4				
Total		N	60	60				
		%	100.00%	100.00%				
Activity Structure	lec/lis	N	28	22	12.988	8	0.112	0.329
		%	46.70%	36.70%				
	lec/per	N	2	0				
		%	3.30%	0.00%				
	dir/lis	N	0	1				
		%	0.00%	1.70%				
	ask/ans	N	1	5				
		%	1.70%	8.30%				
	ev/per	N	6	15				
		%	10.00%	25.00%				
	obs/per	N	1	2				
		%	1.70%	3.30%				
	ev/cop	N	13	7				
		%	21.70%	11.70%				
	obs/cop	N	4	5				
		%	6.70%	8.30%				
	NA/tran	N	5	3				
		%	8.30%	5.00%				
Total		N	60	60				
		%	100.00%	100.00%				
Mode	writing	N	0	9	13.219	7	0.067	0.332
		%	0.00%	15.00%				
	aural	N	34	28				
		%	56.70%	46.70%				
	verbal	N	1	0				
		%	1.70%	0.00%				
	wr-au	N	0	1				
		%	0.00%	1.70%				
	re-au	N	1	0				
		%	1.70%	0.00%				
	au-re	N	2	1				
		%	3.30%	1.70%				
	ver-au	N	20	18				
		%	33.30%	30.00%				
	au-ver	N	2	3				
		%	3.30%	5.00%				
Total		N	60	60				
		%	100.00%	100.00%				
Language Content	Academic	N	5	3	1.509	2	0.47	0.112
		%	8.30%	5.00%				
	Light	N	0	1				
		%	0.00%	1.70%				
	Dense	N	55	56				
		%	91.70%	93.30%				
Total		N	60	60				
		%	100.00%	100.00%				
Language of Instructional (Teacher)	L2	N	54	32	20.745	3	<0.001	0.416
		%	90.00%	53.30%				
		S.R	1.7	−1.7				
	L1-L2	N	0	5				
		%	0.00%	8.30%				
		S.R	−1.6	1.6				
	L2-L1	N	1	6				
		%	1.70%	10.00%				
		S.R	−1.3	1.3				
	NA	N	5	17				
		%	8.30%	28.30%				
		S.R	−1.8	1.8				
Total		N	60	60				
		%	100.00%	100.00%				
Language of Instructional (Student)	L2	N	23	21	0.144	1	0.705	0.035
		%	38.30%	35.00%				
	NA	N	37	39				
		%	61.70%	65.00%				
Total		N	60	60				
		%	100.00%	100.00%				

S.R. = Standardized Residual.
